# Visual ModuleOrganizer: a graphical interface for the detection and comparative analysis of repeat DNA modules

**DOI:** 10.1186/1759-8753-5-9

**Published:** 2014-03-28

**Authors:** Sebastien Tempel, Emmanuel Talla

**Affiliations:** 1 Aix-Marseille Université, CNRS, LCB, UMR 7283, 13009 Marseille, France

**Keywords:** Compressed suffix tree, Maximal repeats, Repeat modules, Graphical interface

## Abstract

**Background:**

DNA repeats, such as transposable elements, minisatellites and palindromic sequences, are abundant in sequences and have been shown to have significant and functional roles in the evolution of the host genomes. In a previous study, we introduced the concept of a repeat DNA module, a flexible motif present in at least two occurences in the sequences. This concept was embedded into *ModuleOrganizer*, a tool allowing the detection of repeat modules in a set of sequences. However, its implementation remains difficult for larger sequences.

**Results:**

Here we present *Visual ModuleOrganizer*, a Java graphical interface that enables a new and optimized version of the *ModuleOrganizer* tool. To implement this version, it was recoded in C++ with compressed suffix tree data structures. This leads to less memory usage (at least 120-fold decrease in average) and decreases by at least four the computation time during the module detection process in large sequences. *Visual ModuleOrganizer* interface allows users to easily choose *ModuleOrganizer* parameters and to graphically display the results. Moreover, *Visual ModuleOrganizer* dynamically handles graphical results through four main parameters: gene annotations, overlapping modules with known annotations, location of the module in a minimal number of sequences, and the minimal length of the modules. As a case study, the analysis of FoldBack4 sequences clearly demonstrated that our tools can be extended to comparative and evolutionary analyses of any repeat sequence elements in a set of genomic sequences. With the increasing number of sequences available in public databases, it is now possible to perform comparative analyses of repeated DNA modules in a graphic and friendly manner within a reasonable time period.

**Availability:**

*Visual ModuleOrganizer* interface and the new version of the *ModuleOrganizer* tool are freely available at: http://lcb.cnrs-mrs.fr/spip.php?rubrique313.

## Background

Repeated sequences (*e.g.* transposable elements, minisatellites,...) are present in all living organisms studied until date [1]. They are evolutionary conserved sequences and have been shown to have a significant functional importance [2]. Recent studies show the role of transposable elements in the evolution of host genomes [3-5], including transposable elements domestication (neogene), exaptation, and transcription regulators [1,6,7]. A number of tools has been described for the search of repeated elements in a genome. However, most of them (*RepeatMasker*[8], *Censor*[9], and *ISFinder*[10]) are BLAST-like tools that detect repeats (such as transposable elements) using a library of consensus sequences. Except for phylogeny analysis, there are few bioinformatic tools (VISTA [11], GATA [12], GraphDNA [13], Recon [14] and DomainOrganizer [15]) that facilitate the analysis of relationships and variations between the copies of a given family of repeats [16,17].

In a previous study, we developped *ModuleOrganizer* that indexed all maximal repeats (MR) of sequences via a suffix tree in order to detect conserved modules within the repeated sequences [18]. Indeed, the algorithm recursively associates two MR if the spacer between them is smaller than the size of the largest maximal repeat and if the edit distance between spacers of all occurrences is not greater than the size of the smallest maximal repeat. The MR association yields to the formation of modules under restrictions defined by the user such as the minimal length of the module (*MinSizeModule*) and the minimal number sequences (*MinSequences*) [18].

Although the previous version of the *ModuleOrganizer* tool can efficiently detect repeated modules within sequences of smaller sizes (<100 Kbp), its implementation with larger sequences remains problematic. This is often because memory usage becomes bottleneck. Since *ModuleOrganizer* command lines are the limiting factors for its use, a graphical interface should be useful for launching and analyzing *ModuleOrganizer* results. To overcome these difficulties, we propose an optimized version of the *ModuleOrganizer* software with its corresponding graphical interface called *Visual ModuleOrganizer*.

## Implementation

Since the previous algorithm was written in C language, the new version of *ModuleOrganizer*[18] was first completely recoded in C++ which drives better memory management. Then, as using a standard suffix tree [19] to compute and store all maximal repeats (MR) leads to a high memory usage during the process of *ModuleOrganizer*, a data structure based on the compressed suffix tree Välimäki [20] was applied to the new algorithm version.

Therefore, the final re-implementation of the new version contains options of the previous algorithm such as ‘search of palindromic modules’, ‘search of exact repeats’, ‘search of truncated modules’, ‘creation of a classification tree file’, ‘search in a minimal number of sequences’ and ‘association distance between MR’. In addition, new options were also developed including: the *‘-limit integer’* option which stops the *ModuleOrganizer* run process after the detection of *integer* modules, and the *‘-f MinSizeMR’* option that selects MR of equal or greater size than *MinSizeMR* bp before the formation of the module. This option decreases the *ModuleOrganizer* run time but have little effects on the sensitivity/specificity of module detection (data not shown). Finally, the new option *‘-p m’* prints each *m* minute the approximative progress of the software.

As the input, *ModuleOrganizer* needs FASTA file containing the nucleotide sequence(s) (*input-file1*, mandatory). It can also use a FASTA reference (*input-file2*, optional) file. This reference sequence file must contain a unique sequence. The *‘-REF input-file2’* option limits the module detection to MR present in both input and reference sequences.

*ModuleOrganizer* creates one mandatory output file (named ‘Module File’) that contains the list of detected modules in a tabular format as follows:

where *START*1, *START*2, *START*3 and *START*4 (*END*1, *END*2, *END*3 and *END*4) correspond to the start (end) location of modules within the sequences. *x* and *y* represent the identification number of modules. *nb*_*sequence* is the number of sequences in which the module is present and *orientation* gives the orientation of the module in sequences (letter ‘*d*’ for the direct strand and letter ‘*c*’ for the reverse strand).

If the input file contains three or more sequences, *ModuleOrganizer* creates an Unweighted Pair Group Method with Arithmetic Mean (UPGMA)-based tree from the matrix of presence/absence of modules in sequences. This tree is then saved as a second output file, with ‘upgma’ extension name. The *‘-MR’* option writes out (in a tabular format) the list of detected MR (see Additional file 1). The option *‘-SVG’* allows the creation of an optional output file in Scalar Vector Graphics (SVG) format, a XML-based vector language that grants modifications with any Scalar Vector Graphics-enabled image processing tool.

The new *ModuleOrganizer* version was successfully compiled and tested on Linux 64 bits, Windows7 32/64 bits, and MacOsX 64 bits. The *Visual ModuleOrganizer* interface was coded and compiled with Java version 1.6.

## Results and discussion

### Data-processing improvements

**Figure 1:**
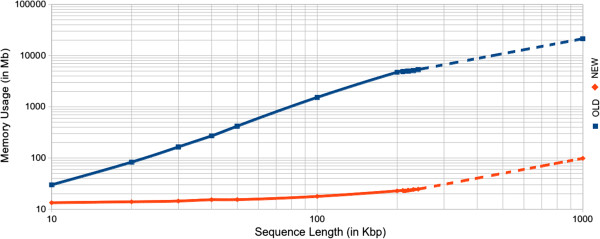
**Memory usage comparison between old and new versions of the ModuleOrganizer algorithm.** The blue (red) line corresponds to the old (new) version of the algorithm. The size range of the sequences is from 10 Kbp to 1000 Kbp. Experienced and expected results are displayed with plain and dotted lines, respectively.

During the run process of the previous algorithm, positions of the selected MR are first copied in the computer memory space before the building of all potential modules. By consequence, this method might lead to high memory usage and therefore slows down the detection process. In order to improve the efficiency of *ModuleOrganizer* memory usage, the new algorithm directly reads the MR positions through the compressed suffix tree data structure. As shown in Figure 1, this change (from suffix tree to compressed suffix tree) dramatically decreases the memory usage of *ModuleOrganizer*, particulary for large sequences. Indeed, in the previous and the new version, artificial random sequences from 10 to 240 Kbp exhibit a memory usage of 29 to 5326 Mb and 13 to 25 Mb, respectively. As shown in Figure 1, the expected required memory space for a 1 Mbp genome should be about 21 Gb with the previous program (>8 Gb of RAM memory for a standard computer) while it should only require 98 Mb with the new version. Interestingly, a typical running process with the new version of *ModuleOrganizer* reduces the required memory space by at least 120 times in average when compared to the old version. Moreover, the new *ModuleOrganizer* version is faster than the previous one, especially for sequences greater than 200 Kbp (Additional file 2). Indeed, for a 240-Kbp sequence, the new tool is 4.8 times faster than the previous version (57 and 277 minutes, respectively). For larger sequences (about 1 Mb), the expected running time do not exceed four hours with the new algorithm of *ModuleOrganizer*.

### The visual ModuleOrganizer graphical interface

For an user-friendly *ModuleOrganizer* and results easily handle, a *Visual ModuleOrganizer* interface was created and divided into two main areas as described below.

#### The visual ModuleOrganizer tool parameters

**Figure 2:**
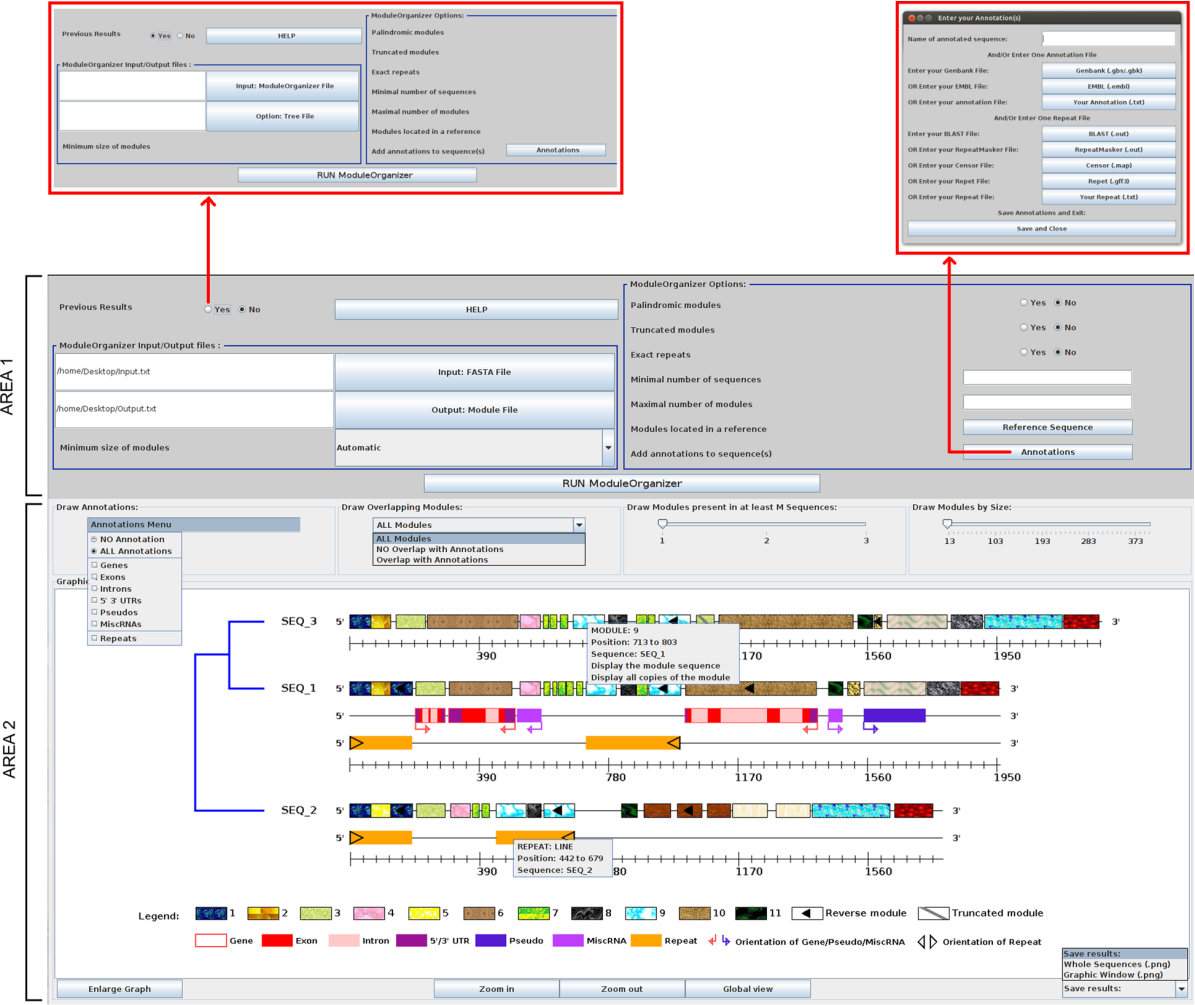
**Screenshoot of the Visual ModuleOrganizer graphical interface.** Text legends of the module textures, genomic objects and repeat annotations are shown under the graphic display.

Through *Visual ModuleOrganizer*, *ModuleOrganizer* parameters (Area 1 in Figure 2) can be used in two different ways: (i) *ModuleOrganizer* is launched by selecting ‘No’ on the ‘Using Previous Results’ button; or (ii) previous results from *ModuleOrganizer* are displayed by selecting ‘Yes’ with the same button.

In the first case, the two first mandatory parameters are the ‘Input: FASTA File’ button, which selects the input file, and the ‘Output: Module File’, in which *ModuleOrganizer* results are written and displayed by the *Visual ModuleOrganizer* interface. The third mandatory parameter (*e.g.MinSizeModule*) is the minimal size of the module, which is chosen from a list (Figure 2). Therefore, a module is detected and selected by the algorithm if its size is equal or longer than *MinSizeModule*. By default (with ‘Automatic’ size), *ModuleOrganizer* proposes to set *MinSizeModule* to the minimal value of *x* such that it does not exists a word of size *x* in the sequence(s).

In addition to the three mandatory parameters, *Visual ModuleOrganizer* has the seven optional parameters. The three first parameters (‘Palindromic modules’, ‘Truncated modules’, and ‘Exact repeats’) provide binary choices and were described in the previous version [18]. Four others were computed in the new version of *ModuleOrganizer*. ‘Minimal number of sequences’ (*e.g.MinSequences*) and ‘Maximal number of modules’ (*e.g.MaxModules*) options require an integer value *x*, and limit the detection of modules in at least *x* sequences and to a maximal number of modules *x* in the whole set of sequences, respectively. The option ‘Module located in a reference’ refers to detected modules located in the reference sequence as well as in the mandatory ‘Input FASTA File’ sequences. The last option, labeled ‘Annotations’, opens a new frame (see upper right frame in Figure 2) that allows the user to add genomic annotations from Genbank [21], EMBL [22], AB-BLAST [23], or NCBI-BLAST [24] and/or repeat annotations from RepeatMasker [8], Censor [9], and Repet [25] formats within the graphical interface. It is noteworthy that each annotation should be added independently for each sequence. Interestingly, the user can add its own annotation in a tabular format.

In the second case, when the button ‘Yes’ from ‘Using Previous Results’ is selected (see upper left frame in Figure 2), *ModuleOrganizer* parameters become invisible. They are replaced by a mandatory parameter ‘Input: ModuleOrganizer File’ and two optional parameters labeled ‘Option: Tree file’ from an upgma-based tree and ‘Annotations’ as described above. Both ‘Input: ModuleOrganizer File’ and ‘Option: Tree file’ use the result file created from a previous *ModuleOrganizer* run.

In both cases, once all the parameters are selected, the user launches the *ModuleOrganizer* algorithm with the ‘RUN ModuleOrganizer’ button. Detailed information about parameters and graphical options are available with the ‘HELP’ button (Additional file 3).

#### Graphical display and optional parameters

A graphical display of the *ModuleOrganizer* results is illustrated in Figure 2 (Area 2). By default, each sequence (*e.g.* SEQ_3) is represented by two lines: one corresponds to a graduated ruler along the sequence (from 5’ to 3’) and the other consists of modules (boxes with different textures). Identical modules are displayed with the same texture to facilitate intra- and inter-sequence comparisons. A reverse module is indicated by a black triangle (*e.g.* module 9 in SEQ_3) while a truncated module (*e.g.* module 3 in SEQ_3) is shown by a dark grey diagonal line within the texture. When an user clicks on a graphical element, a menu with the detailed information (nature of the genetic object and its location) is displayed (*e.g.* a detailed information is shown for a repeat on SEQ_2 in Figure 2). Similar pop-up menu (*e.g.* module 9 in SEQ_3) allows the user to display the nucleotide sequence of a particular or all copies of repeated modules (with their co-ordinates along each sequence), which can be useful for further analysis. It is noteworthy that the sequence order is based on upgma-based tree (by default) instead of alphabetical name order.

Above the graphical panel, four options are provided allowing the user to dynamically add or remove elements (from results or annotations) in the graph. The ‘Draw annotations’ option displays or removes annotations on graph based on the selected item thanks to the ‘Annotations Menu’ menu. When genomic or repeat annotations are displayed, one or two additional lines, corresponding to genetic objects and repeat annotations from ‘Annotations’ files, are added between the module and the ruler lines (Figure 2, see SEQ_1 and SEQ_2 with respectively two and one additional lines, in the context of ‘ALL annotations’ item). The genomic annotations include six different items: ‘Genes’, ‘Exons’, ‘Introns’, ‘5’ ‘3’ UTRs’, ‘Pseudos’ (pseudogenes), ‘MiscRNAs’; that can be selected independently. Their orientations are shown by an arrow shape while repeat annotation orientations are indicated by a black triangle. The ‘NO Annotation’ item removes all annotations.

The ‘Draw Overlapping Modules’ menu displays or removes detected modules based on their overlap or not with visible annotations (genomic or repeat). ‘All Modules’ item draws modules wherever the annotations (Figure 2, Area 2), while ‘Overlap with Annotations’ and ‘NO Overlap with Annotations’ (see Additional file 4), respectively draws and removes the modules that overlap the annotation positions.

‘Draw Modules present in at least M Sequences’ and ‘Draw Modules by Size’ sliders display modules located within a minimal number of sequences and with a minimal length, respectively. Minimun and maximum values of the two sliders are automatically taken from the *ModuleOrganizer* process with the ‘M Sequences’ ranges from 1 to total number of sequences and the ‘Size’ ranges from minimal and maximal length of the detected modules.

Under the graph, four buttons facilitate the modification of the graphical view: ‘Zoom in’ and ‘Zoom out’ buttons increase and decrease by a factor 2 the graph width, respectively. ‘Global view’ button adjusts the graph width (including the graph elements) according to the largest sequence. The last button, labeled ‘Enlarge Graph’, (or ‘Reduce Graph’ after a click on it) removes the parameter area (Figure 2, Area 1) (or displays it) from the interface.

Finally, the ‘Save results’ combo-list saves the graph (whole graph or viewed graph in a PNG format) for external use. All graphical options can be changed and associated at any moment and the graph dynamically displays the elements based on the user choices. Detailed information about parameters and graphical options are available through the ‘HELP’ button (Additional file 3).

### A case study: the FoldBack4 transposable element family of Drosophila melanogaster

FoldBack elements are a family of transposable elements described in *Drosophila melanogaster*. Structurally, the members of this dispersed repetitive family have long inverted terminal repeats and a central loop between the repeats. The lengths of these repeats and loops vary from element to element [26,27]. The inverted repeats of all the family members are homologous [27] and carry a peculiar organization of sequences with highly conserved complex sequences at the termini [28]. Several families of FoldBack, including the FoldBack4 (FB4) family, are known to be non-autonomous transposable elements [26,27].

**Figure 3:**
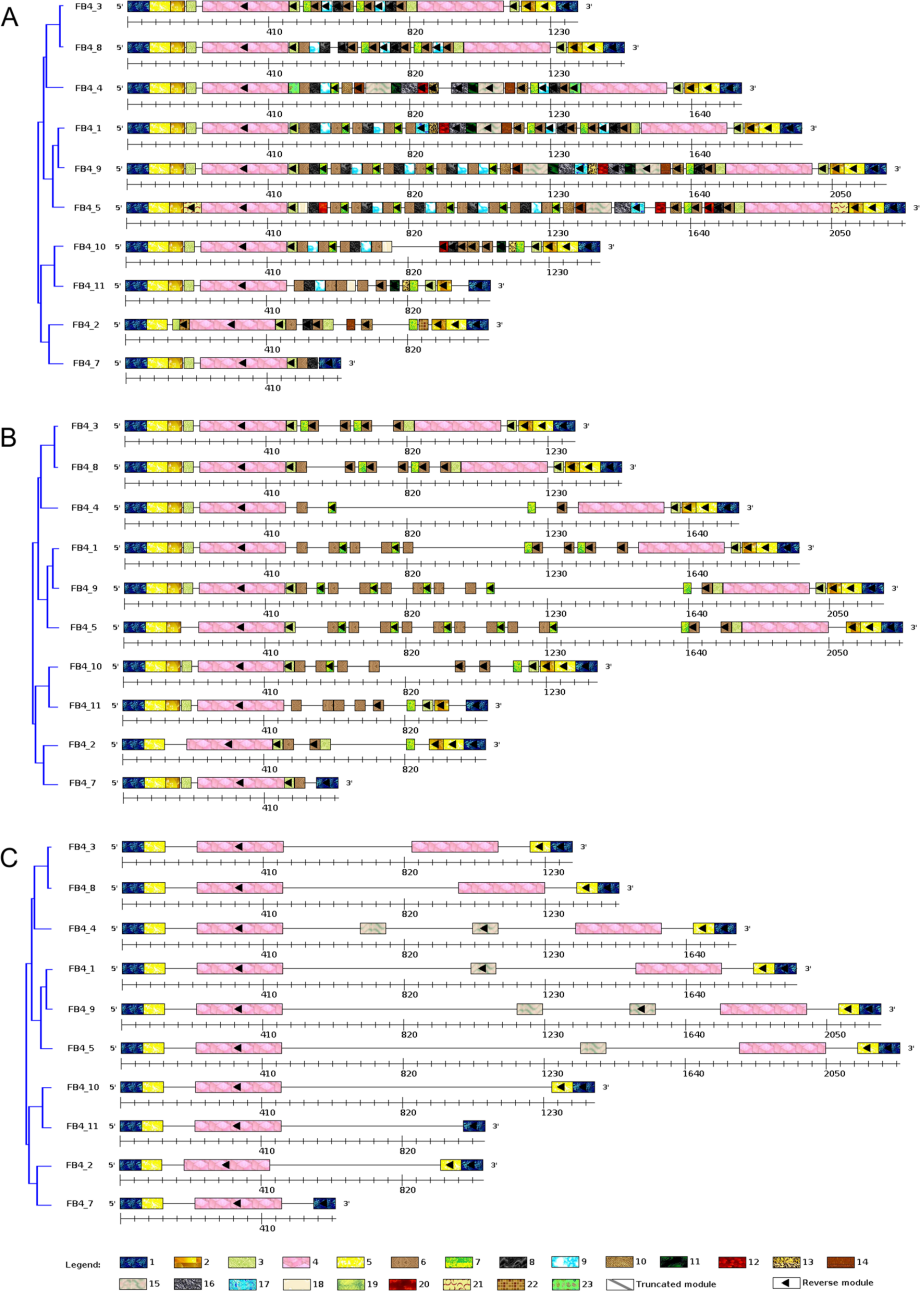
**Identification and comparative analysis of repeat DNA modules in FoldBack4 sequences using Visual ModuleOrganizer.** From the ten FoldBack4 sequences, a *MinSizeModule* of 25, ‘Palindromic modules’ and ‘Truncated modules’ options, the *ModuleOrganizer* algorithm detects 23 modules. Graphical displays of the results: **(A)** default graphical options, **(B)** ‘Draw Modules present in at least M Sequences’ slider sets to 9 and **(C)** ‘Draw Modules by Size’ slider sets to 58 bp.

For this study, 10 FB4 sequence elements ranging from 627 to 2266 bp were chosen. These elements are generally highly variable in their internal sequence, including numerous insertions, deletions, and repetitions, but share consensus palindromic extremities in all their copies because they are necessary for the transposition [28]. With a *MinSizeModule* settled to 25 bp, ‘Palindromic modules’ and ‘Truncated modules’ options selected, the *ModuleOrganizer* algorithm discovered 23 modules (Figure 3A). Palindromic structures of the FB4 sequences are described by modules 1-5 that should correspond to Terminal Inverted Repeat (TIR). Internal sequences are mainly composed of the modules 8-10 which are repeated in tandem, looking like minisatellites. Those are often present in the internal sequence of non-autonomous transposable elements [1,28]. According to the module composition, the upgma-based tree clusters the FB4 sequences in 4 distinct groups: Group1 = FB4_3, FB4_8, and FB4_4; Group2 = FB4_1, FB4_9, and FB4_5; Group 3 = FB4_10 and FB4_11; Group4 = FB4_2 and FB4_7, allowing inter- and intra-groups comparison of the detected modules. Indeed, the reverse occurrence of modules 3 and 4 were deleted in FB4_2 and FB4_7 (from Group4) and reverse modules 2-5 were absent in FB4_10 and FB4_11 (from Group3). These findings clearly suggest that partial deletions of these palindromic structures would impair the transposition of these FB4 sequences.

Through the *Visual ModuleOrganizer* interface, the ‘Draw Modules present in at least M Sequences’ slider was settled to 9, allowing only the display of modules present in at least nine of the ten FB4 sequences. As a result, the palindromic modules (module 1 to 5) and the module 9 from the internal sequence are still displayed (Figure 3B), indicating that those palindromic modules are evolutionary conserved within the FB4 family and might be essential for the transposition. This observation also puts forward that the ‘Draw Modules present in at least M Sequences’ option can be useful for comparative analysis (insertions, deletions, repetitions, rearrangements,...) of modules in a given set of a sequence family. In a similar way, when the ‘Draw Modules by Size’ slider is set to 58 (*e.g* the displayed modules are equal or longer than 58 bp), only modules 1, 4, 5 and 15 are displayed in Figure 3C, therefore allowing the identification of large conserved modules.

Altogether, the case study of FB4 spotlights the ability of *Visual ModuleOrganizer* for comparative analysis of highly complex and variant repeat structures in a given set of sequences. These complex repeat features include biological known repeat structures (palindromes, minisatellites,...) that are usually not observable by standard analysis tools such as VISTA [11], GATA [12], GraphDNA [13], and Recon [14]. Indeed, it has been shown that these software which combine multiple alignment with graphical tools fail to retrieve a good organization of the non-autonomous elements for a typical family such as FB4 [18]. Therefore, *ModuleOrganizer* remains the sole algorithm that is able to identify distinct structural repeats such as duplicated, palindromic and truncated modules, allowing the user to infer putative functional role of these modules.

## Conclusion

We have described *Visual ModuleOrganizer*, a novel graphical interface with a new optimized implementation of the *ModuleOrganizer* tool. The key features of these tools are: (1) detection of modules within larger sequences and with efficient low memory usage; (2) user-friendly handling of *ModuleOrganizer* thanks to a graphical interface; and (3) dynamic graphical parameters that tune the visualization of the results based on the user needs. In addition, *Visual ModuleOrganizer* will be useful to investigate evolutionary and comparative analysis (modules insertions, deletions, rearrangements,...) from all type of DNA repeats (transposable elements, CRISPR, minisatellites,...). *ModuleOrganizer* remains applicable in principle to any set of nucleic sequences sharing some similarities and for which a multiple alignment fails to correctly retrieve the architecture of conserved blocks in the sequences. With the increasing number of sequence data available in biological databases, these features in the *Visual ModuleOrganizer* interface clearly provide new opportunities for inter- and intra-sequence comparative analysis of repeat DNA modules in an easy, user-friendly manner within a reasonable time.

## Abbreviations

CRISPR: Clustered regularly interspaced short palindromic repeats; FB4: FoldBack4; MR: Maximal repeats; RAM: Random access memory; SVG: Scalar vector graphics; UPGMA: Unweighted pair group method with arithmetic mean.

## Competing interests

The authors declare that they have no competing interests.

## Authors’ contributions

ST and ET conceived the study, analyzed the results and wrote the paper. ST performed the computational implementations. All the authors read and approved the final manuscript.

## Supplementary Material

Additional file 1 **Example maximal repeat (MR) output file in a tabular format.** Each file shows six columns: the maximal repeat word, the identification number in the suffix tree, the MR size, the occurrence number, the number of sequences where the MR is present and the list of start positions. Click here for file

Additional file 2 **Run time comparison between old and new versions of ModuleOrganizer.** The blue (red) line represents running time process observed with old (new) version of *ModuleOrganizer*. The size range of the sequences is from 10 Kbp to 1000 Kbp. Experienced and expected results are displayed with plain and dotted lines, respectively. Click here for file

Additional file 3 Screenshoot of the HELP interface. Click here for file

Additional file 4 **Module display when the ‘NO Overlap with Annotations’ item is selected.** Some modules (e.g. Module 1 and 2) became invisible when the ‘NO Overlap with Annotations’ item is selected. Click here for file
