# Supplementary figures and images for: Visual ModuleOrganizer: a graphical interface for the detection and comparative analysis of repeat DNA modules
Source: Mob DNA. 2014 Mar 28;5:9. doi: 10.1186/1759-8753-5-9 (PMC4022104; doi:10.1186/1759-8753-5-9)

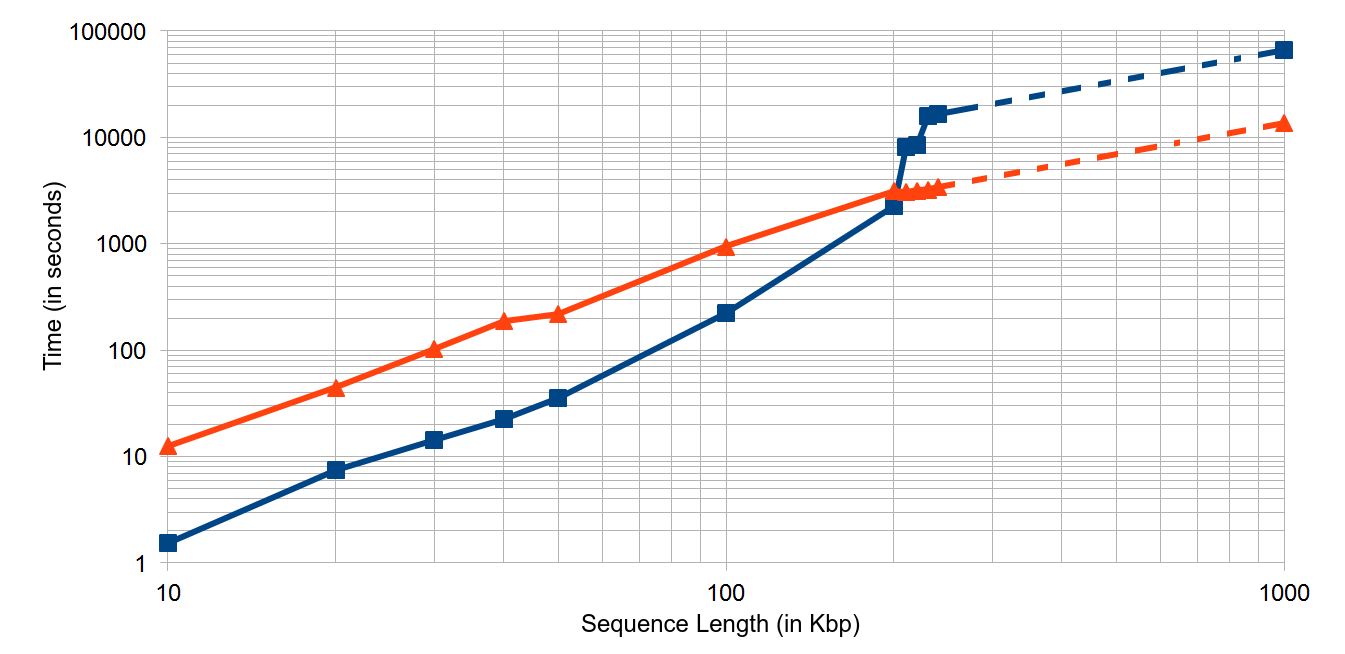

Supplement: Additional file 2 — Run time comparison between old and new versions of ModuleOrganizer. The blue (red) line represents running time process observed with old (new) version of ModuleOrganizer. The size range of the sequences is from 10 Kbp to 1000 Kbp. Experienced and expected results are displayed with plain and dotted lines, respectively. [file 1759-8753-5-9-S2.png]

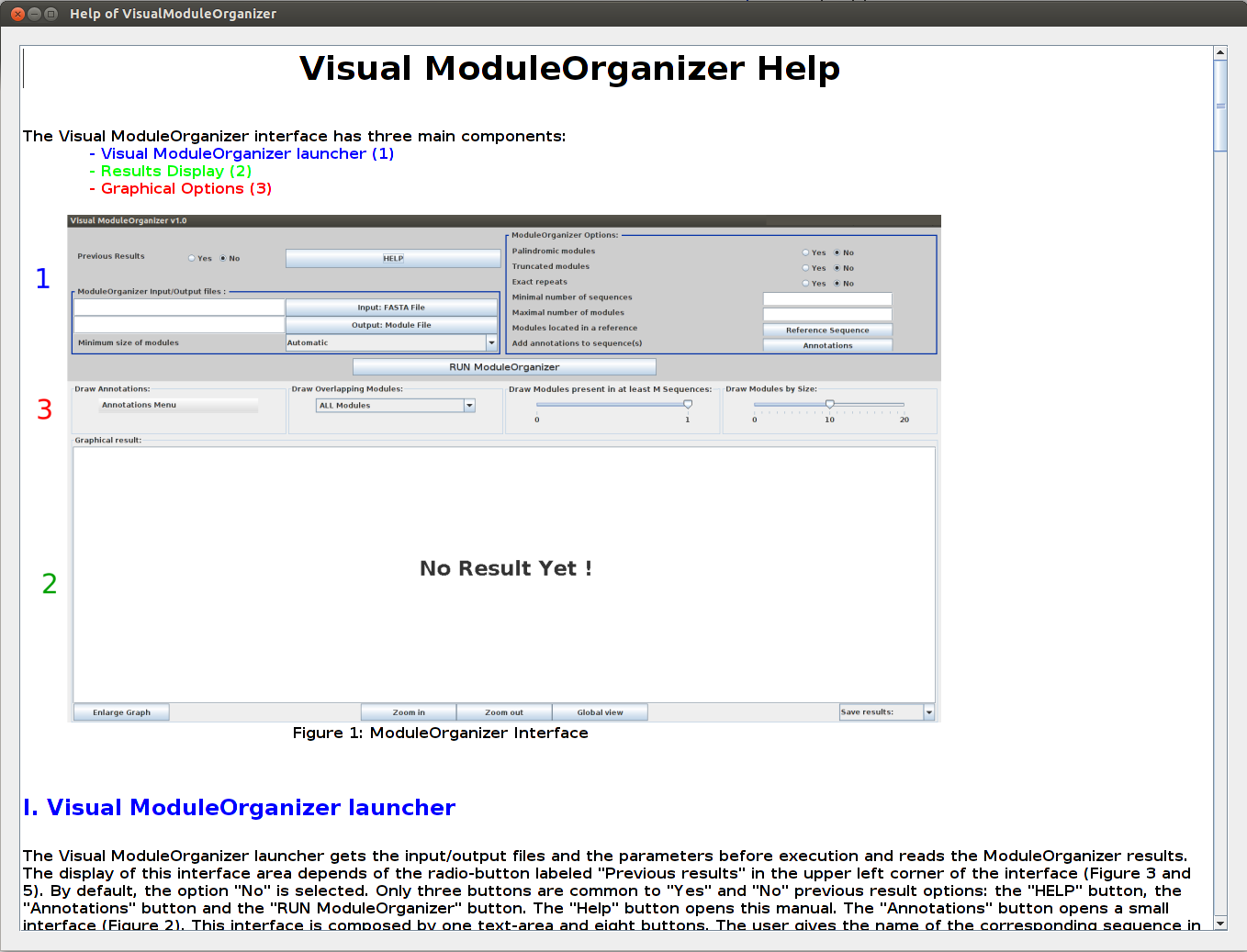

Supplement: Additional file 3 — Screenshoot of the HELP interface. [file 1759-8753-5-9-S3.png]

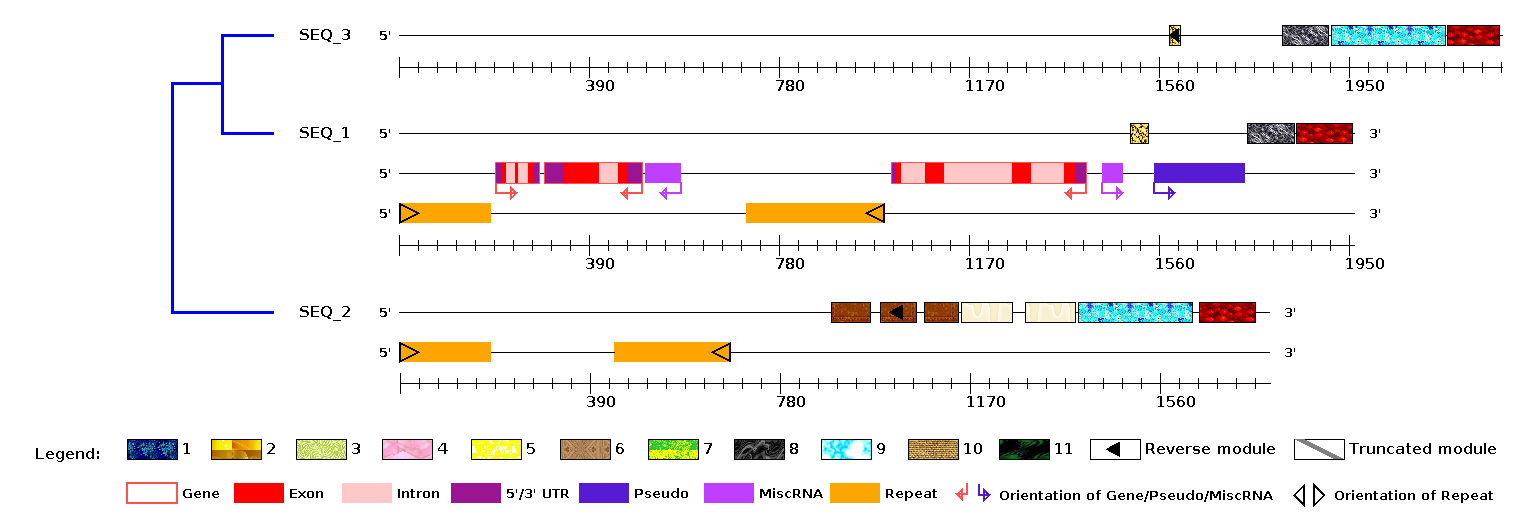

Supplement: Additional file 4 — Module display when the ‘NO Overlap with Annotations’ item is selected. Some modules (e.g. Module 1 and 2) became invisible when the ‘NO Overlap with Annotations’ item is selected. [file 1759-8753-5-9-S4.png]
